# Integrated Clinical Decision Support Systems Promote Absolute Cardiovascular Risk Assessment: An Important Primary Prevention Measure in Aboriginal and Torres Strait Islander Primary Health Care

**DOI:** 10.3389/fpubh.2017.00233

**Published:** 2017-09-04

**Authors:** Veronica Matthews, Christopher P. Burgess, Christine Connors, Elizabeth Moore, David Peiris, David Scrimgeour, Sandra C. Thompson, Sarah Larkins, Ross Bailie

**Affiliations:** ^1^The University of Sydney, University Centre for Rural Health – North Coast, Lismore, NSW, Australia; ^2^Top End Health Service, Northern Territory Government, Darwin, NT, Australia; ^3^Aboriginal Medical Services Alliance Northern Territory, Alice Springs, NT, Australia; ^4^The George Institute for Global Health, Sydney, NSW, Australia; ^5^Spinifex Health Service, Tjuntjuntjara, WA, Australia; ^6^Western Australian Centre for Rural Health, University of Western Australia, Geraldton, WA, Australia; ^7^College of Medicine and Dentistry, James Cook University, Townsville, QLD, Australia

**Keywords:** cardiovascular disease, risk assessment, Indigenous health, prevention, primary health care

## Abstract

**Background:**

Aboriginal and Torres Strait Islander Australians experience a greater burden of disease compared to non-Indigenous Australians. Around one-fifth of the health disparity is caused by cardiovascular disease (CVD). Despite the importance of absolute cardiovascular risk assessment (CVRA) as a screening and early intervention tool, few studies have reported its use within the Australian Indigenous primary health care (PHC) sector. This study utilizes data from a large-scale quality improvement program to examine variation in documented CVRA as a primary prevention strategy for individuals without prior CVD across four Australian jurisdictions. We also examine the proportion with elevated risk and follow-up actions recorded.

**Methods:**

We undertook cross-sectional analysis of 2,052 client records from 97 PHC centers to assess CVRA in Indigenous adults aged ≥20 years with no recorded chronic disease diagnosis (2012–2014). Multilevel regression was used to quantify the variation in CVRA attributable to health center and client level factors. The main outcome measure was the proportion of eligible adults who had CVRA recorded. Secondary outcomes were the proportion of clients with elevated risk that had follow-up actions recorded.

**Results:**

Approximately 23% (*n* = 478) of eligible clients had documented CVRA. Almost all assessments (99%) were conducted in the Northern Territory. Within this jurisdiction, there was wide variation between centers in the proportion of clients with documented CVRA (median 38%; range 0–86%). Regression analysis showed health center factors accounted for 48% of the variation. Centers with integrated clinical decision support systems were more likely to document CVRA (OR 21.1; 95% CI 5.4–82.4; *p* < 0.001). Eleven percent (*n* = 53) of clients were found with moderate/high CVD risk, of whom almost one-third were under 35 years (*n* = 16). Documentation of follow-up varied with respect to the targeted risk factor. Fewer than 30% with abnormal blood lipid or glucose levels had follow-up management plans recorded.

**Conclusion:**

There was wide variation in CVRA between jurisdictions and between PHC centers. Learnings from successful interventions to educate and support centers in CVRA provision should be shared with stakeholders more widely. Where risk has been identified, further improvement in follow-up management is required to prevent CVD onset and reduce future burden in Australia’s Indigenous population.

## Introduction

Health inequities between Aboriginal and Torres Strait Islander (respectfully referred to as Indigenous) and non-Indigenous Australians are well documented (1, 2) and are a legacy of colonization, disempowerment and ongoing racial, social and economic inequality (3). It has been estimated that continued inequality accounts for between one-third and one-half of the 10-year life expectancy gap between Indigenous and non-Indigenous people (2, 4), highlighting the importance of addressing the social determinants of health and ensuring equity of access to quality health care.

Highly preventable chronic diseases contribute most to the higher rate of poor health and premature death experienced by Aboriginal and Torres Strait Islander people. Cardiovascular disease (CVD), largely driven by the combined effect of several modifiable risk factors such as smoking and obesity, is the leading contributor accounting for one-fifth of the health gap (1). In addition to improving social and economic determinants of health, effective CVD prevention, through regular screening and early intervention, would make a significant contribution to reducing the health gap and disease burden within the Indigenous population (5).

Health promotion, prevention, and early treatment services are a key component of Australia’s primary health care (PHC) system. Access to PHC for Aboriginal and Torres Strait Islander people is through community-controlled health centers, government-operated community health centers, and private general practitioners (GPs), with some variation across diverse geographies. Aboriginal and Torres Strait Islander community-controlled centers and some government centers operating in predominantly Indigenous communities offer models of comprehensive PHC providing access to doctors, nurses, allied health, social and emotional wellbeing professionals, and medical specialists. Service size, however, varies depending on remoteness, with visiting services a feature of remote locations.

A recent national initiative, “*Better cardiac care for Aboriginal and Torres Strait Islander people*,” outlines priority action areas to address inequities in cardiovascular health service delivery between Indigenous and non-Indigenous people (6). Priority actions are staged across the disease continuum and include cardiovascular risk assessment (CVRA) as a key aspect of primary prevention, along with practitioner follow-up and intervention for those identified at risk, such as pharmacotherapy and ongoing culturally appropriate support to facilitate lifestyle modification (5).

Absolute CVRA is a screening and management process intended for use by PHC practitioners to calculate the probability of a cardiovascular event within 5 years, taking into account the synergistic effect of multiple risk factors that may be present (7). The risk calculator takes account of age, sex, systolic blood pressure, smoking status, levels of total and high-density lipoprotein cholesterol, and presence of diabetes (7).

Despite the importance of CVRA as a screening and early intervention tool, few studies have reported its use within the Indigenous PHC sector or in the broader Australian PHC setting. This reflects the lack of national and jurisdictional data on CVRA and PHC services in general (6). In 2012, the Northern Territory (NT) government implemented a large-scale strategy to strengthen chronic disease prevention in Indigenous communities that included regular CVRA data collection and reporting and the roll-out of an automated CVRA calculator within the electronic medical record system used by government health centers (8). In 2015, a similar calculator was introduced into the *Communicare* electronic medical record system used by many Aboriginal and Torres Strait Islander community-controlled health centers in the NT and other jurisdictions.

This study examines variation in documented CVRA for adults with no prior diagnosis of chronic disease as a primary prevention strategy in Indigenous PHC centers across four Australian jurisdictions (2012–2014). We also report on the proportion of Indigenous people found with elevated risk and the proportion that had subsequent follow-up actions documented.

## Materials and Methods

This is a retrospective cross-sectional study of preventive care clinical audits undertaken by 97 Indigenous PHC centers participating in the Audit and Best Practice for Chronic Disease (ABCD) project. The ABCD project is a research-based continuous quality improvement (CQI) initiative that has operated on a national scale since 2005, co-designing best practice clinical audit tools (covering different aspects of comprehensive PHC delivery) and processes with relevant stakeholders (9). The majority of participating services within the ABCD program are community-controlled or government-operated centers predominantly serving Indigenous communities. The preventive care CQI process was designed to enable participating health centers assess the level of adherence to best practice guidelines and assess organizational systems to support prevention and early detection of chronic disease. The audits are conducted by local staff trained in the use of ABCD tools and processes.

Clients included in a preventive care audit are those aged ≥15 years with no recorded diagnosis of diabetes, hypertension, coronary heart disease, rheumatic heart disease, or chronic kidney disease, who have been resident in the community for ≥6 months within the last year (10). Where the eligible population numbers 30 or less, the audit protocol recommends inclusion of all records. For 30 or more eligible clients, the protocol provides guidance on a sufficient number of randomly selected records to achieve 90–95% confidence of the sample representing the service population. Samples were stratified by age and gender.

Given the value of risk assessment as a primary prevention measure and high rates of premature CVD in Indigenous people, the audit tool incorporates assessment against best practice guidelines related to CVRA delivery. Current national guidelines recommend CVRA be provided to Indigenous adults aged between 35 and 74 years who are not known to have CVD or to be at clinically determined high risk (7). For NT Indigenous residents, the CVRA age criterion has been lowered to 20 years due to local prevalence of early onset CVD (11). Low, moderate, and high risks correspond to <10, 10–15, and >15% probability of a cardiovascular event within the next 5 years as determined by the type of calculator used in the assessment. As no CVRA algorithm has been validated in the Indigenous Australian population, current calculators underestimate the risk, failing to consider historical context and the consequential socio-economic disadvantage and premature CVD prevalent within the population (12). Following the precedent set within New Zealand’s CVRA guidelines for the Maori population, the NT PHC standard treatment (CARPA) manual included an upward risk adjustment of 5% on the Framingham algorithm for the NT Indigenous population (11, 13).

**Figure 1:**
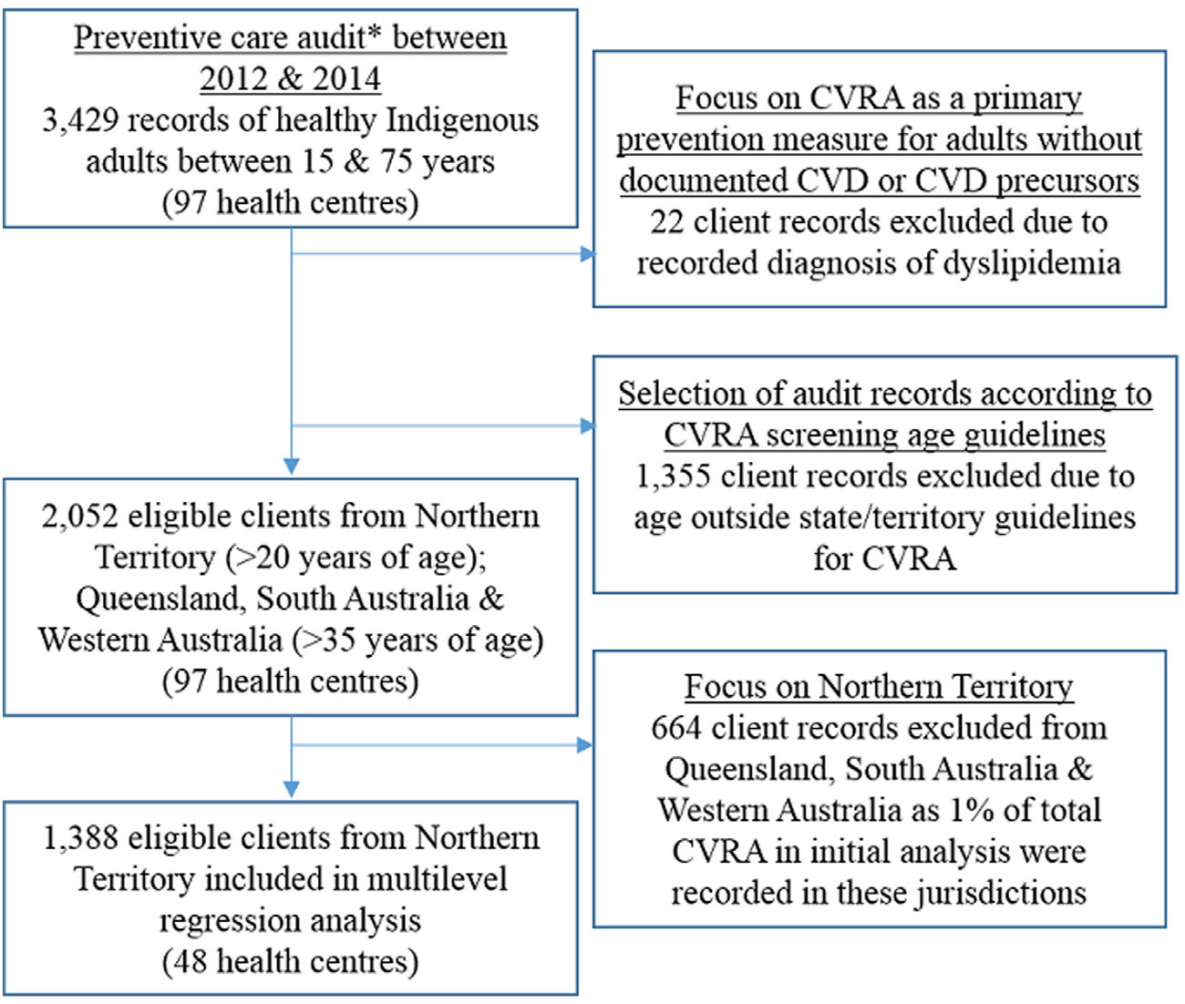
Inclusion criteria of Aboriginal and Torres Strait Islander client records to examine cardiovascular risk assessment (CVRA) as primary prevention strategy. *A preventive care audit excludes clients with a record of diabetes, hypertension, coronary heart disease, rheumatic heart disease, and chronic kidney disease.

A subset of the preventive care audit data was used for this study to examine variation in documented CVRA as a primary prevention strategy for Indigenous adults with no previous documentation of chronic disease (Figure 1). We used the most recent preventive care audit from each of the 97 centers conducted between 2012 and 2014. While the audit tool includes clients with a diagnosis of dyslipidaemia, we excluded these records (*n* = 22) given it is a prominent risk factor for CVD and may influence clinical judgments with respect to a patient’s background and calculating absolute risk (14). Almost 90% of clients with a record of dyslipidaemia did not have a record of CVRA within the last 24 months. Our “healthy cohort” criteria have therefore excluded individuals eligible for CVRA according to national guidelines, such as people with diabetes under the age of 60 years without microalbuminuria or people with stages 1 and 2 chronic kidney disease. De-identified clinic records of over 2,000 healthy Indigenous adults were included in the analysis. In addition to demographic information (age and sex), the audit recorded whether individuals received an adult health check and a CVRA within the last 24 months and the calculated CVD risk level. Other information collected included relevant risk factor documentation [yes/no for smoking status, body mass index, waist circumference, urinalysis, blood pressure, and blood glucose and lipid levels] and follow-up actions for abnormal findings. Health center factors such as location, population size, governance, and length of participation in the ABCD program were also recorded.

Our main outcome measure was the proportion of eligible clients who had documented CVRA. We used client and health center level information to determine independent factors associated with CVRA. Secondary outcomes for those identified at moderate/high risk were the proportion that had documented follow-up management plans and brief interventions. It was not possible to assess the level of pharmacotherapy intervention as this information was not captured within the audit tool.

Summary statistics was used to describe variation in CVRA across health centers and jurisdictions, the number of adults with elevated risk and documented level of follow-up. Cross-jurisdictional information has been aggregated where there were counts less than five. Given the hierarchical nature of the data (clients within health centers), multilevel mixed effects logistic regression models were used (health center variable was treated as a random effect with random intercept) to quantify the variation in CVRA attributable to health center and client level factors. Because a large majority of CVRA was recorded in NT centers, we restricted the regression analysis to this jurisdiction. As most centers were located in remote areas, we excluded location as a predictor variable. We calculated odds ratios to measure the unadjusted and adjusted associations between independent factors and CVRA (adjusting for year of audit). In a step-wise fashion, we included significant health center (Model A) then client variables (Model B) from the unadjusted analyses and measured the proportional change in variance to determine the amount of variation attributable to the different levels. Potential interactions were checked for significance. Statistical associations were considered significant if the *p*-value was <0.007 (Bonferroni correction). Analysis was completed using STATA software, version 14.

## Results

The majority (92%) of PHC centers were located in remote or very remote areas, and 84% were government operated (Table 1). There were 2,052 eligible Indigenous clients aged between 20 and 75 years with almost equal numbers of males and females. Acute care was the primary reason for attendance for 49% of clients. There was wide variation across jurisdictions and health centers in the documentation of risk factors used to assess CVD risk (Table 2). NT centers had higher documentation of client smoking status, blood pressure, and lipid profile (essential measures for the Framingham algorithm) compared to other jurisdictions. Overall, 23% of eligible clients had CVRA documented and almost all of the assessments occurred in the NT (Table 3).

**Table 1:** Characteristics of primary health care (PHC) centers and clients.

		NT	QLD	SA/WA	Total
Number of PHC centers		48	42	7	97
		***n* (%)**	***n* (%)**	***n* (%)**	***n* (%)**
Location^a^	Non-remote	1 (2)	2 (5)	5 (71)	8 (8)
Governance	Remote/very remote	47 (98)	40 (95)	2 (29)	89 (92)
	Community-controlled	11 (23)	1 (2)	4 (57)	16 (16.5)
	Government	37 (77)	41 (98)	3 (43)	81 (83.5)
Service population (*n*)	≤500	26 (54)	22 (52.4)	1 (14)	49 (50.5)
	501–999	8 (17)	9 (21.4)	3 (43)	20 (20.6)
	≥1,000	14 (29)	11 (26.2)	3 (43)	28 (28.9)
Continuous quality improvement (CQI) cycles completed	Baseline	10 (21)	4 (10)	2 (29)	16 (16.5)
	1 or 2 cycles	13 (27)	16 (38)	4 (57)	33 (34)
	>3 CQI cycles	25 (52)	22 (52)	1 (14)	48 (49.5)

Number of client records		1,388	509	155	2,052
Age (years)	20 to <35	904 (65)	NA	NA	904 (44)
	35 to <45	283 (20)	255 (50)	59 (38)	597 (29)
	45 to <75	201 (15)	254 (50)	96 (62)	551 (27)
Sex	Male	677 (49)	255 (50)	77 (50)	1,009 (49)
	Female	711 (51)	254 (50)	78 (50)	1,043 (51)
Reason for last attendance	Health check	200 (14)	60 (11.8)	70 (45)	330 (16)
	Acute care	686 (49)	273 (53.6)	42 (27)	1,001 (49)
	Immunization	102 (7)	85 (16.7)	4 (3)	191 (9)
	Others	400 (29)	91 (17.9)	39 (25)	530 (26)

*^a^Location based on the Australian Standard Geographical Classification system*.

*NT, Northern Territory; QLD, Queensland; SA, South Australia; WA, Western Australia*.

**Table 2:** Primary health care (PHC) center documentation of risk factors used for cardiovascular risk assessment (median % and range).

	Northern Territory	Queensland	South Australia/Western Australia
Number of PHC centers	48	42	7
Risk factors: smoking status	73% (25–95)	54% (0–100)	86% (56–100)
Body mass index/waist circumference	80% (17–100)	27% (0–94)	77% (63–100)
Blood pressure (BP)	93% (54–100)	87% (29–100)	92% (78–100)
Blood lipid profile^a^	72% (31–100)	33% (0–81)	39% (0–86)
Blood glucose	88% (50–100)	68% (0–100)	83% (72–100)
Urinalysis	71% (28–100)	31% (0–81)	11% (0–100)
Smoking status/BP/lipid profile^b^	61% (21–94)	24% (0–81)	33% (0–86)

*^a^A lipid profile includes total cholesterol, high-density lipoprotein, low-density lipoprotein, and triglycerides*.

*^b^Minimum level of risk factor documentation required for estimates using the Framingham algorithm*.

**Table 3:** Documented delivery of cardiovascular risk assessment (CVRA) by primary health care (PHC) centers (mean % and total number), by jurisdiction (2012–2014).

	Northern Territory	Queensland/South Australia/Western Australia	Total
Number of PHC centers	48	49	97
Median % (range) CVRA	38% (0–86)	0% (0–36)	19% (0–86)
Number of eligible clients	1,388	664	2,052
CVRA recorded	471 (34%)	7 (1%)	478 (23%)

**Figure 2:**
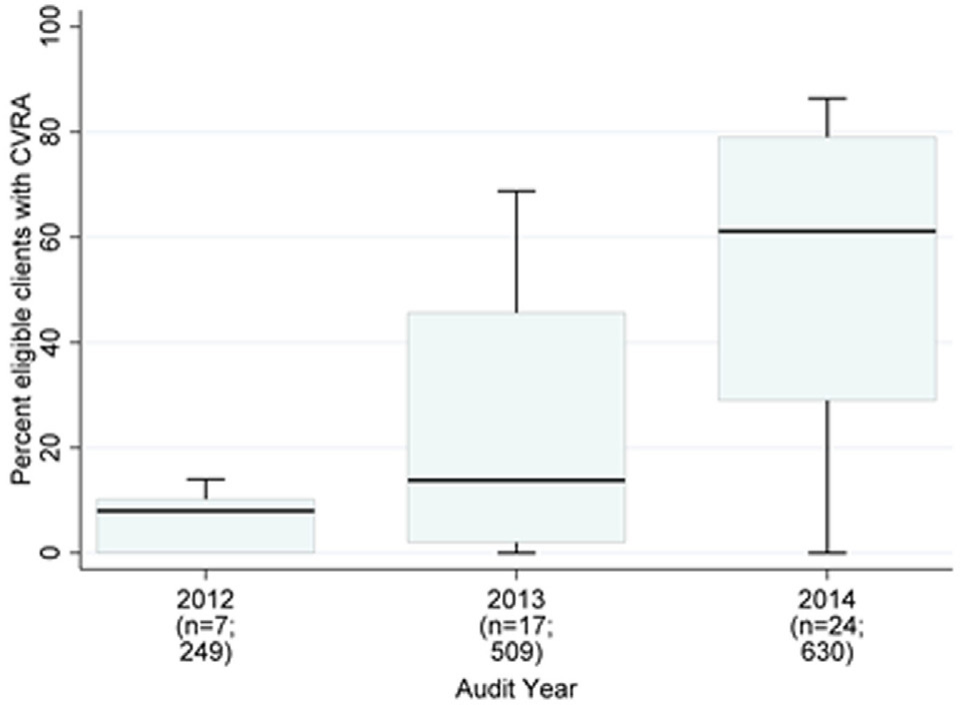
Northern Territory health center mean percent of clients with documented cardiovascular risk assessment (CVRA) (2012–2014).

Due to the audit exclusion criteria and local CVRA guidelines, the NT cohort (*n* = 1,388) had a larger proportion (65%) of young adults (<35 years). Focusing on this jurisdiction, there was a clear trend of improvement in the mean proportion of PHC center clients documented as receiving CVRA; however, wide variation persisted across years (Figure 2). Health center factors accounted for 48% of the variation (Table 4, Model A). Clients were more likely to have documented CVRA if they attended government-operated centers (distinguished by the availability of automated CVRA calculators within their electronic patient information systems, Model A: OR 21.1; 95% CI 5.4–82.4; *p* < 0.001), and if they had an adult health check (Model A: OR 3.9; 95% CI 2.8–5.4; *p* < 0.001). There was no significant interaction between governance and provision of health checks. Client factors did not appreciably explain any further variation, although adults aged ≥45 years were more likely to have CVRA compared to the youngest age group (Model B: OR 2.0; 95% CI 1.3–3.2; *p* = 0.003).

**Table 4:** Multilevel regression analysis—cardiovascular risk assessment (CVRA) in Northern Territory primary health care centers, 2012–2014 (*n* = 48 centers; 1,388 clients).

Fixed effects		Unadjusted analysis			Model A			Model B		
		Odds ratio	95% CI	*p*-value	Odds ratio	95% CI	*p*-value	Odds ratio	95% CI	*p*-value
**Outcome is client record of CVRA**										
Audit year	2012	1.00	(reference)		1.00	(reference)		1.00	(reference)	
	2013	4.72	(0.88–25.2)	0.07	3.05	(0.74–12.5)	0.099	3.02	(0.72–12.7)	0.13
	2014	24.7	(4.94–123)	<0.001	18.8	(4.72–74.4)	<0.001	20.4	(5.02–82.8)	<0.001
Predictors										
**Health center factors**										
Governance	Other centres	1.00	(reference)		1.00	(reference)		1.00	(reference)	
	Government operated	36.9	(7.36–185)	<0.001	21.1	(5.38–82.4)	<0.001	21.6	(5.39–86.9)	<0.001
Service population (*n*)	≥1,000	1.00	(reference)		1.00	(reference)		1.00	(reference)	
	>500 to <1,000	1.97	(0.37–10.4)	0.42	0.98	(0.26–3.77)	0.98	0.96	(0.25–3.79)	0.96
	≤500	14.7	(4.20–51.3)	<0.001	2.31	(0.78–6.86)	0.13	2.37	(0.78–7.18)	0.13
Provided adult health check	No	1.00	(reference)		1.00	(reference)		1.00	(reference)	
	Yes	4.00	(2.87–5.57)	<0.001	3.89	(2.81–5.41)	<0.001	3.94	(2.83–5.49)	<0.001
Duration of participation in Audit and Best Practice for Chronic Disease continuous quality improvement	Baseline	1.00	(reference)							
	1–2 cycles	1.09	(0.30–4.01)	0.90						
	≥3 cycles	8.82	(1.50–51.8)	0.016						
**Client factors**										
Age (years)	≥20 to <35	1.00	(reference)					1.00	(reference)	
	≥35 to <45	1.52	(1.04–2.22)	0.032				1.55	(1.05–2.31)	0.029
	≥45 to <75	1.90	(1.23–2.93)	0.004				2.00	(1.27–3.15)	0.003
Sex	Male	1.00	(reference)							
	Female	0.80	(0.60–1.07)	0.13						
Random effects (intercepts)		Empty model (audit year only)								
Health center [variance (SE)]		2.99	(0.86)		1.56	(0.46)		1.63	(0.47)	
PCV (% explained variance)					48%			46%		

*PCV, proportional change in variance; significance level p < 0.007*.

Of the NT clients who received CVRA, 11% (*n* = 53; 95% CI: 8–14%) were found to be at moderate/high risk of a cardiovascular event in the next 5 years (Table 5). Thirty percent of adults with elevated risk were under 35 years (*n* = 16; Table 5) and 64% (*n* = 34) had ≥3 modifiable risk factors documented (Table 6). The main risk factors recorded were abnormal lipid levels (*n* = 43 at risk clients), being overweight (*n* = 38), and smoking (*n* = 33; Table 6). In terms of follow-up action documented, less than one-third of clients with abnormal blood lipid or glucose levels had a management plan recorded (with a scheduled repeat measurement), 82% (*n* = 27) of smokers and 71% (*n* = 27) of overweight clients had documentation of a brief intervention or referral (Table 6).

**Table 5:** Number (%) of Northern Territory clients with documented cardiovascular risk assessment (CVRA) and risk level, by age group.

Age group (years)	20 to <35	35 to <45	45 to <75	Total	
		
	*n* (%)	*n* (%)	*n* (%)	*n* (%)	
CVRA completed	299 (33)	99 (35)	73 (36)	471 (34)	
**Documented risk level**					**95% CI^a^**
Risk level not recorded	14 (5)	7 (7)	2 (2.7)	23 (5)	
High/moderate	16 (5)	14 (14)	23 (31.5)	53 (11)	(8–14%)
Low	269 (90)	78 (78)	48 (65.8)	395 (84)	(80–87%)
CVRA recorded as not done	605 (67)	184 (65)	128 (64)	917 (66)	
Total	904	283	201	1,388	

*^a^95% confidence intervals calculated for population proportion of risk level estimates*.

**Table 6:** Documentation of risk factors and follow-up interventions in Northern Territory clients with elevated risk (*n* = 53).

	*n* (%)	

Number of risk factors present		
<3 risk factors	19 (36)	
≥3 risk factors	34 (64)	
	**Risk factor**	**Follow-up action**
	***n* (%)**	***n* (%)**
Tobacco use^b^	33 (62)	27 (82)
High body mass index (BMI)^a,b^	38 (72)	27 (71)
Abnormal blood pressure^a,c^	10 (19)	6 (60)
Abnormal blood glucose^a,c^	31 (59)	9 (29)
Abnormal lipids^a,c^	43 (81)	11 (26)

*^a^High BMI ≥ 25; abnormal blood pressure: systolic pressure ≥140 mmHg and/or the diastolic pressure ≥90 mmHg; abnormal blood glucose: ≥5.5 mmol; abnormal blood lipids: low-density lipoprotein >2.0 mmol/L or high-density lipoprotein <1 mmol/L or triglycerides >1.5 mmol/L*.

*^b^Follow-up action—brief intervention or referral for relevant lifestyle modification (smoking cessation/weight management)*.

*^c^Follow-up action—documented management plan including repeat test schedule to monitor levels and for high blood pressure and lipid readings, referral to doctor for assessment and potential medication control*.

## Discussion

This study provides original data on CVRA as an important CVD primary prevention activity for adults with no prior chronic disease diagnosis in the Aboriginal and Torres Strait Islander PHC sector. CVRA is particularly important in the generally healthy population as adults with known major chronic illnesses such as diabetes are at higher risk of CVD, regardless of the other risk measurements included in CVRA. Our finding that 23% of eligible clients had documented CVRA is lower than in similar studies reporting inadequate levels of screening (15, 16) due to our exclusion of clients with chronic disease, regardless of severity. The NT had substantially higher level documentation of CVRA than other jurisdictions and of those assessed at moderate/high risk, 30% were under the age of 35 years.

There have been similar findings within the Australian general practice sector where it has been reported that GPs: may not routinely calculate absolute risk for the general population; focus treatment on individual risk factors; and lack the data necessary for calculation of absolute risk (especially lipid screening) (17, 18). Lack of risk factor recording is also an issue in the current study, where in some jurisdictions, the minimal level of risk factor documentation in client records required for CVRA calculation (using the Framingham algorithm: smoking status, blood pressure, and lipid profile) was under 35%. Despite significant government funding for tackling smoking initiatives within the Indigenous community, there remains a high level of variation in recording of smoking status, particularly in Queensland. Participating health centers in Queensland were predominantly government-operated and not representative of the community-control sector. The latest national Key Performance Indicator (nKPI) report (December 2014) shows Aboriginal and Torres Strait Islander community-controlled health centers in Queensland documenting smoking status for 83% of their clientele (19).

Despite the recording of individual risk factors in the health records of many clients attending health centers in the NT, almost all CVRA (*n* = 439) occurred within the government sector, which had automated calculators available within their electronic patient information record systems. As manual calculation of CVRA can be cumbersome, this highlights the value of the automated calculator that was not available within the systems used by the community-control sector during the study period. Automation of CVRA also allowed the NT government to implement three monthly rapid CQI cycles on key performance indicators to continually identify screening and treatment gaps (8). There have also been dedicated educators and CQI facilitators coaching local teams in CVRA and assisting with assessments and identification of clients for recall while located off-site from the PHC centers. Sustained implementation of these higher level system supports differentiates the NT from other jurisdictions where large-scale CQI auditing for preventive care has taken place without improvement in CVRA. Other targeted interventions incorporating electronic decision support tools and CQI processes have also led to increased rates of CVRA coverage across PHC centre service populations in urban, rural and remote locations in Australia and New Zealand (20, 21). Learnings from these successful interventions have the potential to improve screening rates in other areas, and it is likely that coverage rates improved further beyond this study period due to the introduction of the automated calculator within community-control PHC centers.

With the majority of clients in this study presenting for acute care, time and PHC center capacity may be a barrier to investigating CVD precursors in people with no diagnosis of a chronic condition (22). There was an association between CVRA and adult health checks suggesting that the checks are an important initiator of CVRA. Through a rebate system, the Australian Government has encouraged uptake of comprehensive health checks for Indigenous people that includes examination of physical, psychological, and social well-being [Medicare Benefits Schedule (MBS) item 715]. The use of health checks has increased over time and as at December 2014, 46% of regular Indigenous clients aged over 25 years in the NT had a health check within the last 24 months (44% nationally) (19). However, while the health check collects risk factor and biomedical information used to calculate cardiovascular risk, CVRA is not directly specified within the MBS 715 item descriptor. To more immediately address the CVD burden in the Indigenous population, a standalone rebate item for CVRA has been recommended to promote uptake rates beyond that of general health checks (8).

We found a lower proportion of Indigenous people assessed as having moderate/high CVD risk in comparison to that reported for the NT government sector in 2014 (8). This may be partly explained by differences in the study cohorts, with the preventive care audit excluding clients with a record of chronic disease. Based on this criteria, 65% of the NT clients in this study were under the age of 35 years and 15% were 45 years or older. Almost one-third of clients with moderate/high CVD risk were under 35 years, emphasizing the importance of targeted screening for adults younger than the current CVRA national guideline for Indigenous people.

Despite improvements in risk screening, evidence to practice gaps persist regarding follow-up treatment to reduce risk once identified (8, 20). Lack of follow-up has been attributed previously to barriers at various levels of the health system such as lack of time, staff capacity, availability of culturally appropriate referral services, and that practice incentives focus on assessment rather than follow-up (23). The lack of documentation of management plans for abnormal findings demonstrates the challenge of maintaining client continuity of care, particularly in remote contexts. Building client/health provider relationships is an important enabler for sustained engagement required to effect lifestyle change, where responsibility for care is shared between client and practitioner and where health centers work beyond traditional roles to influence social and economic determinants for individuals and their communities (24, 25). Lifestyle modifications such as improving nutrition and exercise and lowering rates of smoking will reduce the current and future burden of disease, not only with respect to CVD but other chronic conditions contributing to the health gap between Aboriginal and Torres Strait Islander and other Australians.

Large-scale examination of CVRA to Indigenous people has been made possible by the centers enrolled in the ABCD CQI program. However, the voluntary nature of their participation limits the generalizability of study findings, with the majority of centers from remote areas in the NT and the government sector in Queensland. In addition, as data are collected from client records, delivery of CVRA and associated follow-up for clients at risk may be underestimated due to poor documentation. However, accurate recording is an essential aspect of care quality and should be addressed as part of CQI processes.

The “*Better cardiac care for Aboriginal and Torres Strait Islander people*” strategy has increased attention on capturing CVRA data on a broad scale, initiating its introduction to the nKPI dataset (26) (although the age criteria begins at 35 years, some 10 years older than clients documented with elevated risk in the NT). A routine data source allows the assessment of “unwarranted” variation in the delivery of key service items, a necessary first step to examine potential health system factors that when leveraged, may enhance consistent, appropriate care on a broad-scale (27, 28). We demonstrate the value of CQI processes in systematically capturing, reporting and reviewing data on the variation in CVRA and follow-up. Systematic collection of data for primary prevention also emphasizes the need for a specific calculator to accurately predict CVD risk for this population.

## Conclusion

Our findings show that there is substantial room for improvement in CVRA and follow-up as an important primary prevention strategy within the Aboriginal and Torres Strait Islander population. Systematic CVRA provides an opportunity for the PHC sector to curb rates of early CVD onset as emphasized by the young “healthy” cohort of Aboriginal and Torres Strait Islander adults documented with elevated risk. Shared learnings from successful system interventions as demonstrated in the NT (integration of automated calculators, CQI processes, and dedicated staff support) have the potential to reduce unwarranted variation and increase rates of screening on a broad scale, enabling early intervention where necessary. Further work is required on improving follow-up of clients identified at risk and facilitating supportive health center–client/community relationships.

## Ethics Statement

Ethics approval was obtained from human research ethics committees (HRECs) in each jurisdiction: Northern Territory HREC-EC00153 & HREC-12-53; New South Wales HREC/11/GWAHS/23; Queensland HREC/11/QTDD/47; South Australia Aboriginal Health Research Ethics Committee 04-10-319; Western Australia Curtin University HR140/2008; WA Country Health Services 2011/27; WA Aboriginal Health Information and Ethics Committee 111-8/05; and University of Western Australia RA/4/1/5051.

## Author Contributions

RB and VM conceived and designed the study. VM analyzed the data, drafted, and revised the paper with important intellectual input from all authors (PB, CC, EM, DP, DS, ST, SL, and RB). RB played a lead role in design and development of the ABCD research project. CC, EM, DS, ST, and SL were co-investigators on the ABCD project. All authors read and approved the final manuscript.

## Conflict of Interest Statement

The authors declare that the research was conducted in the absence of any commercial or financial relationships that could be construed as a potential conflict of interest.

## Abbreviations

ABCD, Audit and Best Practice for Chronic Disease; CQI, continuous quality improvement; CVD, cardiovascular disease; CVRA, cardiovascular risk assessment; GP, general practitioner; nKPI, national Key Performance Indicator; NT, Northern Territory; PCV, proportional change in variance; PHC, primary health care; QLD, Queensland; SA, South Australia; WA, Western Australia.
